# Implication of the Dose of Mineralocorticoid Receptor Antagonist Following Transcatheter Edge-to-Edge Mitral Valve Repair

**DOI:** 10.1016/j.jacasi.2025.03.014

**Published:** 2025-05-27

**Authors:** Teruhiko Imamura, Shuhei Tanaka, Nobuyuki Fukuda, Hiroshi Ueno, Koichiro Kinugawa, Shunsuke Kubo, Masanori Yamamoto, Yuki Izumi, Mike Saji, Masahiko Asami, Yusuke Enta, Shinichi Shirai, Masaki Izumo, Shingo Mizuno, Yusuke Watanabe, Makoto Amaki, Kazuhisa Kodama, Junichi Yamaguchi, Toru Naganuma, Hiroki Bota, Yohei Ohno, Daisuke Hachinohe, Masahiro Yamawaki, Kazuki Mizutani, Toshiaki Otsuka, Kentaro Hayashida, the, OCEAN-Mitral Investigators

**Affiliations:** a Second Department of Internal Medicine, University of Toyama, Toyama, Japan; b Department of Cardiology, Kurashiki Central Hospital, Kurashiki, Japan; c Department of Cardiology, Toyohashi Heart Center, Toyohashi, Japan; d Department of Cardiology, Nagoya Heart Center, Nagoya, Japan; e Department of Cardiology, Gifu Heart Center, Gifu, Japan; f Department of Cardiology, Sakakibara Heart Institute, Tokyo, Japan; g Division of Cardiovascular Medicine, Department of Internal Medicine, Toho University Faculty of Medicine, Tokyo, Japan; h Division of Cardiology, Mitsui Memorial Hospital, Tokyo, Japan; i Department of Cardiology, Sendai Kosei Hospital, Sendai, Japan; j Division of Cardiology, Kokura Memorial Hospital, Kitakyushu, Japan; k Division of Cardiology, St. Marianna University School of Medicine Hospital, Kawasaki, Japan; l Department of Cardiology, Shonan Kamakura General Hospital, Kanagawa, Japan; m Department of Cardiology, Teikyo University School of Medicine, Tokyo, Japan; n Department of Cardiology, National Cerebral and Cardiovascular Center, Suita, Japan; o Division of Cardiology, Saiseikai Kumamoto Hospital Cardiovascular Center, Kumamoto, Japan; p Department of Cardiology Tokyo Woman’s Medical University, Tokyo, Japan; q Department of Cardiology, New Tokyo Hospital, Chiba, Japan; r Department of Cardiology, Sapporo Higashi Tokushukai Hospital, Sapporo, Japan; s Department of Cardiology, Tokai University School of Medicine, Isehara, Japan; t Department of Cardiology, Sapporo Cardiovascular Clinic, Sapporo, Japan; u Department of Cardiology, Saiseikai Yokohama City Eastern Hospital, Kanagawa, Japan; v Division of Cardiology, Department of Medicine, Kinki University Faculty of Medicine, Osaka, Japan; w Department of Hygiene and Public Health, Nippon Medical School, Tokyo, Japan; x Department of Cardiology, Keio University School of Medicine, Tokyo, Japan

**Keywords:** heart failure, hemodynamics, MitraClip, valvular disease

## Abstract

**Background:**

Mineralocorticoid receptor antagonists (MRAs) are integral components of medical therapy for patients with heart failure with reduced ejection fraction. However, implication of MRA dosing in older patients undergoing transcatheter edge-to-edge mitral valve repair (TEER) for secondary mitral regurgitation remains uncertain.

**Objectives:**

The authors aimed to investigate the prognostic impacts of MRA dosing in older patients receiving TEER for secondary mitral regurgitation.

**Methods:**

This study included patients who underwent TEER and were enrolled in the OCEAN (Optimized CathEter vAlvular iNtervention)-Mitral registry. Patients with a left ventricular ejection fraction <50% and secondary mitral regurgitation were selected. The dose-dependent effects of MRA, administered at discharge, on the 2-year composite outcome of all-cause mortality and heart failure hospitalization were evaluated.

**Results:**

A total of 2,026 patients (median age 77 years; 1,287 men) were included and followed for a median 416 days (Q1-Q3: 294-730 days). Post-TEER, the administration of MRA at a dose of ≥12.5 mg/d (ie, any doses of MRA) was independently associated with a lower 2-year cumulative incidence of the primary composite outcome, with an adjusted HR of 0.83 (95% CI: 0.69-0.99; *P =* 0.046). In contrast, higher doses of MRA were not significantly associated with a further reduction in the risk of the primary outcome *(P =* 0.97).

**Conclusions:**

In older patients who underwent TEER for secondary mitral regurgitation caused by systolic heart failure, even a low-dose MRA was associated with improved clinical outcomes compared with no MRA administration. However, further up-titration of the MRA dose did not result in additional improvements in clinical outcomes. (OCEAN-Mitral registry; UMIN000023653)

Clinical outcomes for patients with heart failure with reduced ejection fraction (HFrEF) have markedly improved caused by the advent of guideline-directed medical therapy.^1^, ^2^, ^3^ Notably, recent studies have demonstrated a paradigm shift from conventional renin-angiotensin system inhibitors to angiotensin receptor-neprilysin inhibitors as a primary therapeutic strategy.^4^ Additionally, sodium-glucose cotransporter 2 inhibitors have been shown to reduce mortality and morbidity in patients with chronic heart failure across a wide range of left ventricular ejection fractions (LVEF), independent of the presence of diabetes mellitus.^5^

It is important to recognize the continued relevance of mineralocorticoid receptor antagonists (MRAs), which are well-established disease-modifying medical therapies that decrease cardiovascular mortality and reduce the risk of heart failure hospitalizations in patients with HFrEF.^6^, ^7^, ^8^ Nevertheless, several uncertainties remain regarding MRA-incorporated medical therapy.

First, the feasibility and efficacy of MRA use in older patients with multiple comorbidities, such as chronic kidney disease, have not been fully elucidated.^9^ Second, the optimal dosing of MRAs in real-world clinical practice remains unclear, particularly for older patients, even though current guidelines advocate for up-titration to the maximum dose.^1^, ^2^, ^3^ Third, in patients with significant secondary mitral regurgitation (MR) caused by HFrEF, heart failure symptoms may persist even after the reduction of MR via transcatheter edge-to-edge mitral valve repair (TEER).^10^ The prognostic impact of MRA therapy in this specific cohort has not been adequately studied.

Considering these unresolved issues, we investigated the dose-dependent prognostic impact of MRAs in older patients with significant MR who underwent TEER, using data from a large-scale, multicenter, investigator-driven registry.

## Methods

### Participant selection

This retrospective study was conducted using a prospectively developed, multicenter, investigator-driven OCEAN (Optimized CathEter vAlvular iNtervention)-Mitral registry data set.^11^ The registry compiled clinical data on patients who underwent TEER using the MitraClip system for significant MR between April 2018 and June 2023.

From this cohort, we excluded patients with primary MR, those with an LVEF ≥50%, and those dependent on hemodialysis. The study was registered with the University Hospital Medical Information Network Clinical Trials Registry (UMIN000023653). The research protocol for the creation of the registry database received approval from the Institutional Review Board at each participating hospital, in compliance with the ethical principles outlined in the Declaration of Helsinki.

### Study design

Patients were followed for a period of 2 years after undergoing TEER, with day 0 defined as the date of the TEER procedure. The independent variable was the dose of MRA prescribed following TEER during the index hospitalization. The primary outcome was a composite of all-cause mortality and heart failure–related hospitalizations post-TEER. Hospitalization for worsening heart failure, necessitating intravenous diuretic agents or other related therapies during careful in-hospital monitoring, was determined at the discretion of the attending board-certified cardiologists.

### TEER procedure

The decision to proceed with TEER was made by multidisciplinary local heart valve teams comprising interventional cardiologists, general cardiologists, cardiothoracic surgeons, imaging cardiologists, medical engineers, and other skilled health care professionals. The severity of MR was quantified using the proximal isovelocity surface area method by board-certified echocardiography experts at each institution. Patients were generally required to meet the COAPT (Cardiovascular Outcomes Assessment of the MitraClip Percutaneous Therapy for Heart Failure Patients With Functional Mitral Regurgitation) criteria and be deemed unsuitable for mitral valve surgery.^12^ This decision-making process involved comprehensive discussions with patients and their families, ensuring that informed consent procedures were rigorously followed.

The TEER procedure using the MitraClip system was conducted according to standardized protocols. It was performed under general anesthesia with fluoroscopic and transesophageal echocardiographic guidance. The procedure began with a transseptal puncture via femoral vein access, followed by advancing a 24-F guide catheter into the left atrium. The clip delivery system was then positioned above the origin of the MR jet and further advanced into the left ventricular cavity. The mitral leaflets were engaged, and the clip was temporarily closed to approximate the leaflets. If a satisfactory reduction in MR was achieved, the clip was released. If additional MR reduction was necessary, a second clip was considered based on an assessment of the residual MR and the mean pressure gradient across the mitral valve.

### Follow-up

Patients were closely monitored during the index hospitalization to detect any periprocedural complications. Discharge was considered after confirming the absence of critical procedure-related complications. Following discharge, patients were regularly followed up at the outpatient clinic of each institution or their affiliated centers in a scheduled manner by board-certified cardiologists. Heart failure medications were adjusted based on the patient’s symptoms and test results obtained at each visit, at the discretion of the attending cardiologists.

### Clinical variables obtained

All data utilized in the present study were retrieved from the prefixed OCEAN-Mitral registry database. Clinical variables included pre-TEER characteristics, such as demographic information, comorbidities, laboratory findings, echocardiographic evaluations, and medication records. Echocardiographic assessments were conducted in accordance with the guidelines established by the American Society of Echocardiography.

A COPAT-like profile was defined as absence of all the following criteria: severe left ventricular impairment (left ventricular end-diastolic diameter >70 mm or LVEF <20%), severe tricuspid regurgitation, severe pulmonary hypertension (systolic pulmonary artery pressure >70 mm Hg), and hemodynamic instability with continuous inotropes use.^13^

Procedural data, including anesthesia time, were also collected. Following the procedure, additional echocardiographic data and medication information were obtained, with the dose of MRA defined as an independent variable. Postprocedural data included the duration of hospitalization, in-hospital mortality, and the occurrence of adverse events.

Clinical outcomes were tracked for 2 years after the procedure or until the end of the study period (May 2024). The primary outcome was defined as a composite of all-cause mortality and heart failure admissions during the observation period.

### Statistical analyses

Continuous variables were presented as medians (Q1-Q3), while categorical variables were expressed as numbers and corresponding percentages. Comparisons of continuous variables between the 2 groups were conducted using the Mann-Whitney *U* test, whereas categorical variables were compared using the chi-square test or Fisher exact test. To confirm whether the missing data mechanism was missing completely at random, Little’s MCAR test was conducted.

A cutoff value for the MRA dose after TEER, predictive of the primary outcome, was determined by maximum log-rank statistics. The cumulative incidence of the primary outcome was compared using the log-rank test. Cox proportional hazards regression analysis was performed, following the confirmation of proportional hazards assumption by the Schoenfeld residuals tests, to assess the prognostic impact of MRA dose after TEER on the primary outcome. The findings were adjusted for predefined potential confounders, including age, atrial fibrillation, estimated glomerular filtration rate, use of beta-blockers after TEER, use of renin-angiotensin system inhibitors after TEER, and use of diuretic agents after TEER.

The patient cohort was further stratified into 4 groups based on their MRA dose following TEER to evaluate the detailed prognostic impact of MRA dosing: 1) 0 mg/d; 2) 12.5 mg/d; 3) 25 mg/d; and 4) 37.5 mg/d or greater. The cumulative incidence of the primary outcome was compared among these 4 groups using the log-rank test. The HR was calculated with the 0 mg/d MRA group serving as the reference.

All statistical analyses were conducted using SPSS Statistics software version 23.0 (IBM Corp). A 2-sided *P* value of <0.05 was considered statistically significant for all tests.

## Results

### Patient selection

Of the 3,764 patients who underwent TEER and were registered in the OCEAN-Mitral registry, 1,125 (30%) patients with primary MR, 1,564 (42%) patients with an LVEF of ≥50%, and 229 (6%) patients dependent on hemodialysis were excluded. Consequently, a total of 2,026 patients with secondary MR and an LVEF of <50% were included in the present study. The assumption of missing completely at random was confirmed *(P =* 0.32, chi-square statistics 91170).

### Clinical data before TEER

Clinical data before TEER were displayed in Table 1. Median age was 77 years (Q1-Q3: 70-82 years), and 1,287 (64%) were men. Median plasma B-type natriuretic peptide level was 496 pg/mL (Q1-Q3: 244-949 pg/mL). Approximately one-half of the participants (1,246 [62%]) received MRA before TEER. The median dose of MRA was 25 mg/d (Q1-Q3: 0-25 mg/d).

**Table 1:** Pre-TEER Clinical Characteristics

	Total (N = 2,026)	MRA ≥12.5 mg/d After TEER (Any Doses of MRA) (n = 953)	MRA <12.5 mg/d After TEER (No MRA) (n = 1,073)	*P* Value
Demographics				
Age, y	77 (70-82)	77 (71-82)	77 (70-82)	0.81
Men	1,287 (64)	577 (61)	710 (66)	0.006^a^
Body surface area, m^2^	1.54 (1.40-1.67)	1.53 (1.38-1.67)	1.54 (1.41-1.67)	0.17
Systolic blood pressure, mm Hg	101 (92-115)	100 (90-110)	104 (93-119)	<0.001^a^
Pulse rate, beats/min	72 (64-82)	71 (63-81)	73 (64-83)	0.006^a^
EuroScore II	6.18 (3.79-10.6)	6.14 (3.85-10.1)	6.21 (3.72-11.0)	0.83
Secondary MR	2,026 (100)	953 (100)	1,073 (100)	—
Comorbidity				
Dyslipidemia	1,120 (55)	521 (55)	599 (56)	0.28
Diabetes mellitus	697 (34)	318 (33)	379 (35)	0.44
Atrial fibrillation	1,174 (58)	575 (60)	599 (56)	0.051
History of ventricular tachycardia	303 (15)	155 (16)	148 (14)	0.13
History of stroke	234 (12)	112 (12)	122 (11)	0.43
Inotropes use	356 (18)	196 (21)	160 (15)	<0.001^a^
Laboratory data				
Hemoglobin, g/dL	11.9 (10.6-13.2)	12.0 (10.6-13.2)	11.8 (10.5-13.1)	0.002^a^
Serum albumin, g/dL	3.6 (3.3-4.0)	3.7 (3.3-4.0)	3.6 (3.3-4.0)	0.59
Serum sodium, mEq/L	139 (136-141)	139 (136-141)	139 (136-141)	0.21
eGFR, mL/min/1.73 m^2^	35.4 (23.3-48.9)	37.7 (27.3-50.9)	33.2 (19.5-45.7)	<0.001^a^
Plasma BNP, pg/mL	496 (244-939)	454 (215-793)	525 (260-1,057)	<0.001^a^
Echocardiography data				
LVDD, mm	62 (56-69)	62 (56-68)	62 (56-69)	0.008^a^
LVEF, %	32 (26-39)	32 (26-38)	33 (27-39)	0.001^a^
LVEF <50%	2,026 (100)	953 (100)	1,073 (100)	—
Left atrial volume index, mL/m^2^	74.2 (57.9-97.6)	72.6 (56.7-93.2)	76.1 (59.7-100.3)	0.37
MR regurgitant volume, mL	48 (37-61)	47 (36-61)	48 (38-61)	0.004^a^
Moderate or greater AR	185 (9)	89 (9)	96 (9)	0.42
Moderate or greater TR	627 (31)	270 (28)	357 (33)	0.008^a^
Medication				
MRA use	1,246 (62)	819 (86)	427 (40)	<0.001^a^
MRA dose, mg/d	25 (0-25)	25 (25-25)	0 (0-25)	<0.001^a^
RAS inhibitor use	1,342 (66)	672 (71)	670 (62)	<0.001^a^
ARNI use	271 (13)	194 (20)	77 (7)	<0.001^a^
Beta-blocker use	1,687 (83)	810 (85)	877 (82)	0.078
SGLT2 inhibitor use	579 (29)	441 (46)	138 (13)	<0.001^a^
Diuretic agents use	1,639 (81)	817 (86)	822 (77)	<0.001^a^

Values are median (Q1-Q3) and compared between the 2 groups by Mann-Whitney *U* test or n (%) and compared between the 2 groups by chi-square test or Fisher exact test. Clinical characteristics before transcatheter edge-to-edge mitral valve repair (TEER) were stratified by the dose of mineralocorticoid receptor antagonist (MRA) after TEER.

AR = aortic regurgitation; ARNI = angiotensin receptor neprilysin inhibitor; BNP = B-type natriuretic peptide; eGFR = estimated glomerular filtration rate; LVDD = left ventricular end-diastolic diameter; LVEF = left ventricular ejection fraction; MR = mitral regurgitation; RAS = renin-angiotensin system; SGLT2 = sodium-glucose cotransporter; TR = tricuspid regurgitation.

a *P* < 0.05.

After TEER, patients were categorized into 2 groups based on their MRA dose: 953 (47%) patients received an MRA dose of ≥12.5 mg/d (ie, any doses of MRA), whereas the remaining 1,073 (53%) patients received an MRA dose of <12.5 mg/d (ie, no MRA). Patients who received an MRA dose of ≥12.5 mg/d (ie, any doses of MRA) after TEER had less severe anemia, higher glomerular filtration rate, and lower plasma BNP levels, although the absolute differences in these values were clinically trivial.

### Clinical data after TEER

**Figure 1:**
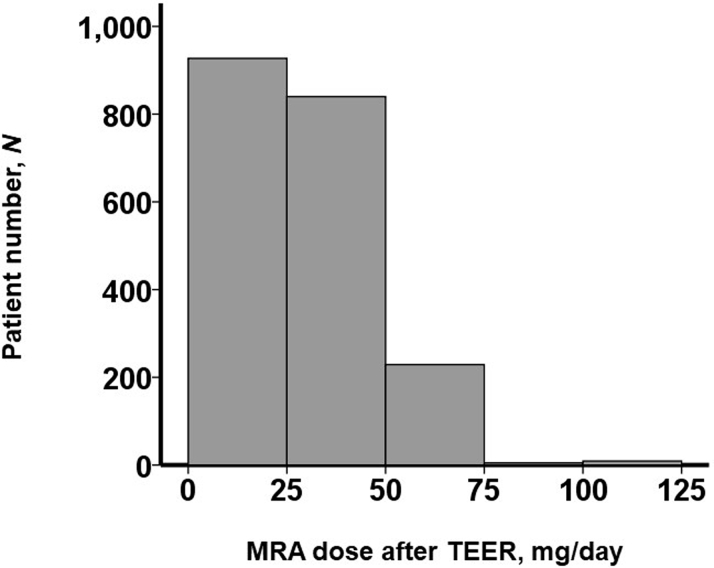
Distribution of MRA Dose After TEER The dose of mineralocorticoid receptor antagonist (MRA) prescribed after transcatheter edge-to-edge mitral valve repair (TEER) distributed widely with approximately one-half of patients receiving no MRA.

Procedural and post-TEER data were displayed in Table 2. All patients had LVEF <50%. Approximately one-half of the participants (953 [47%]) received MRA after TEER. The median dose of MRA was 0 mg/d (Q1-Q3: 0-25 mg/d). Distribution of MRA dose was displayed in Figure 1. The minimum dose of MRA was 12.5 mg/d.

**Table 2:** Clinical Characteristics After TEER

	Total (N = 2,026)	MRA ≥12.5 mg/d After TEER (Any Doses of MRA) (n = 953)	MRA <12.5 mg/d After TEER (No MRA) (n = 1,073)	*P* Value
Procedure data				
Anesthesia time, min	148 (122-186)	147 (123-182)	148 (119-191)	0.076
Procedural time, min	80 (58-110)	76 (58-102)	81 (57-112)	0.007^a^
Device time, min	52 (36-75)	50 (36-70)	53 (35-77)	0.066
Fluoroscopy duration, min	24 (17-36)	26 (19-37)	22 (15-33)	<0.001^a^
Postechocardiography data				
LVDD, mm	60 (54-67)	61 (55-68)	60 (54-66)	0.006^a^
LVEF, %	30 (25-37)	29 (24-36)	31 (25-38)	<0.001^a^
LVEF <50%	2,026 (100)	953 (100)	1,073 (100)	—
Left atrial volume index, mL/m^2^	67.8 (53.4-90.2)	66.4 (52.8-88.3)	69.0 (54.2-93.0)	0.20
Moderate or greater AR	179 (9)	90 (9)	89 (8)	0.43
Moderate or greater TR	456 (23)	193 (20)	263 (25)	0.011^a^
Residual moderate or greater MR	405 (20)	252 (26)	153 (14)	0.39
Mean pressure gradient at mitral valve, mm Hg	2.6 (1.9-3.5)	2.5 (1.8-3.7)	2.7 (2.0-4.0)	0.087
Postmedication				
MRA use	953 (47)	953 (100)	0 (0)	<0.001^a^
MRA dose, mg/d	0 (0-25)	25 (25-50)	—	<0.001^a^
RAS inhibitor use	722 (36)	458 (48)	264 (25)	<0.001^a^
Beta-blocker use	1,319 (65)	824 (86)	495 (46)	<0.001^a^
Diuretic agents use	1,178 (58)	775 (81)	403 (38)	<0.001^a^

Values are median (Q1-Q3) and compared between the 2 groups by Mann-Whitney *U* test or n (%) and compared between the 2 groups by chi-square test or Fisher exact test. Clinical characteristics after TEER were stratified by the dose of MRA, which was administered after TEER.

Abbreviations as in Table 1.

a *P <* 0.05.

The incidence of moderate or greater residual MR was 20%. The mean transvalvular pressure gradient was 2.6 mm Hg (Q1-Q3: 1.9-3.5 mm Hg). Several clinical data significantly differed between the patients with MRA ≥12.5 mg/d (ie, any doses of MRA use) and those with MRA <12.5 mg/d (ie, no MRA use). Notably, a greater number of MRA users had higher rates of utilization of other heart failure medications.

### Clinical outcomes after TEER

During the 2-year observation period following TEER with a median of 416 days (Q1-Q3: 294-730 days), 1,050 (52%) patients experienced the primary outcome (799 deaths and 788 heart failure admissions).

**Figure 2:**
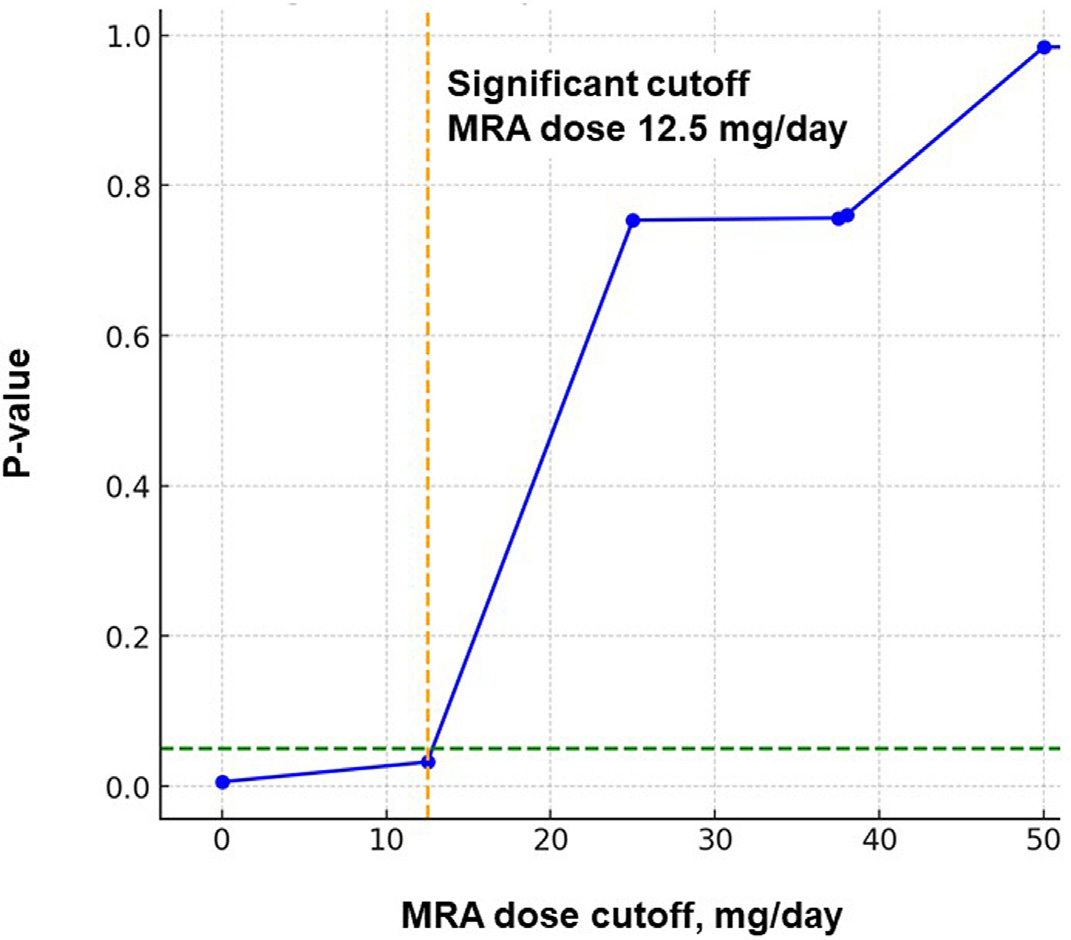
Maximum Log-Rank Statistics of MRA Dose After Transcatheter Edge-to-Edge Mitral Valve Repair for the Primary Outcome The prognostic impact of mineralocorticoid receptor antagonist (MRA) dosing after transcatheter edge-to-edge mitral valve repair on the primary outcome, which was defined as 2-year death or heart failure admission, was analyzed. A cutoff of MRA dose at 12.5 mg/d was calculated as a significant separation of the primary outcomes.

**Figure 3:**
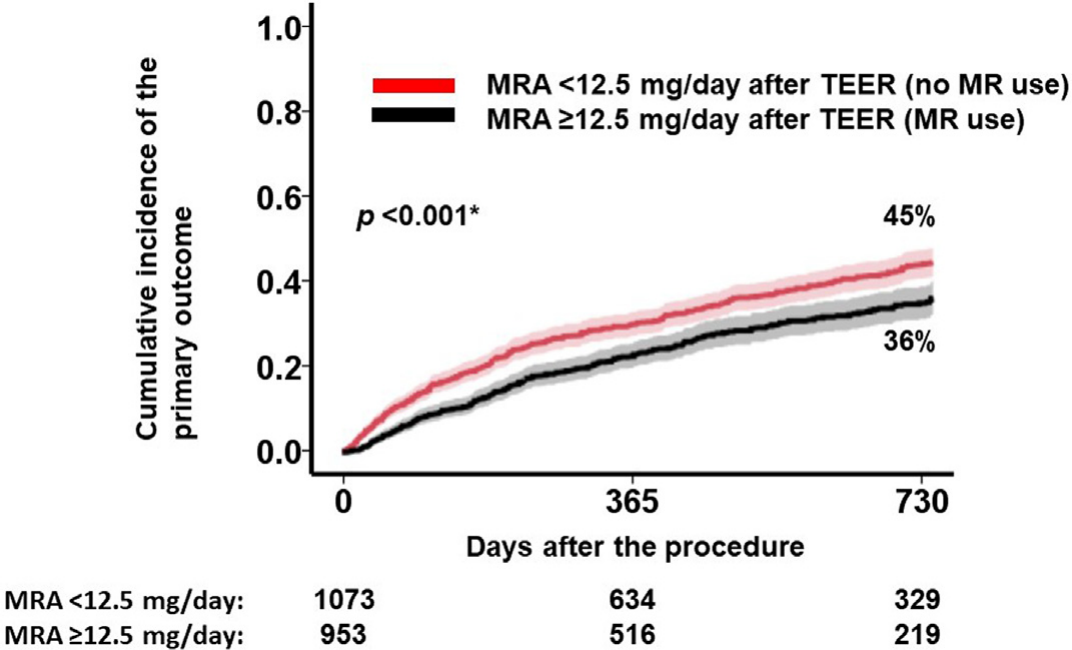
Prognostic Impact of MRA Dose After TEER on the Primary Outcome The cohort was divided into 2 groups by the cutoff of MRA dose: 12.5 mg/d, which was calculated by the maximum log-rank statistics (Figure 2). ∗*P <* 0.05 by log-rank test. Abbreviations as in Figure 1.

The optimal MRA dose after TEER to predict the primary outcome was determined to be 12.5 mg/d (Figure 2). Patients receiving an MRA dose of ≥12.5 mg/d (ie, any doses of MRA) after TEER had a significantly lower cumulative incidence of the primary outcome over the 2-year observation period compared with those receiving <12.5 mg/d (ie, no MRA use) (36% versus 45%; *P* < 0.001) (Figure 3). Administration of an MRA dose of ≥12.5 mg/d post-TEER (ie, any doses of MRA) was independently associated with a reduced risk of the primary outcome, with an HR of 0.83 (95% CI: 0.69-0.99; *P =* 0.046), adjusted for predefined potential confounders including age, atrial fibrillation, estimated glomerular filtration rate, use of beta-blockers post-TEER, use of renin-angiotensin system inhibitors post-TEER, and use of diuretic agents post-TEER.

The presence of moderate or greater residual MR did not have a significant prognostic impact with an HR: 1.13 (95% CI: 0.94-1.35; *P =* 0.21). The mean transvalvular gradient post-TEER was also not significantly associated with the primary outcome with an HR: 1.03 (95% CI: 0.98-1.06; *P =* 0.086). When we further incorporated these 2 variables as additional potential confounders, the adjusted HR was 0.81 (95% CI: 0.65-0.97; *P =* 0.038).

When reviewing the breakdown of the primary outcome, 2-year cumulative mortality was 22% in the MRA ≥12.5 mg/d group compared with 30% in their counterparts (*P <* 0.001). The 2-year cumulative incidence of heart failure admissions was 21% in the MRA ≥12.5 mg/d group vs 25% in those receiving <12.5 mg/d *(P =* 0.032).

### Prognostic impact of stratified MRA dose after TEER

**Figure 4:**
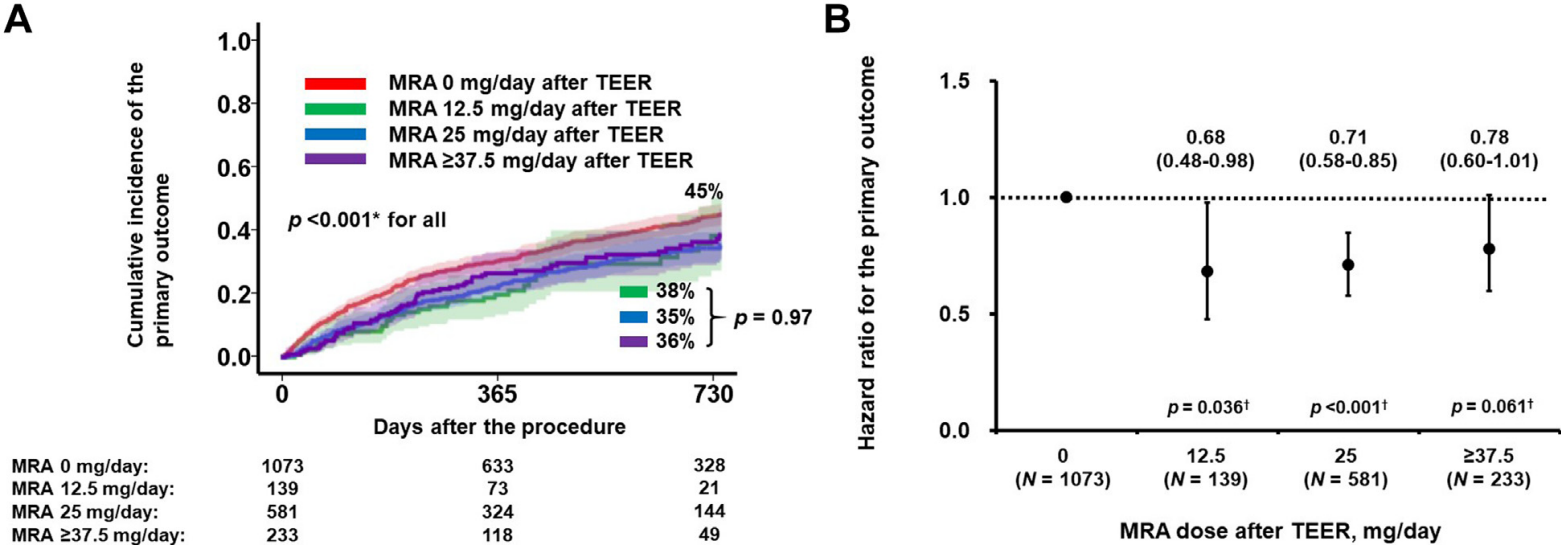
Prognostic Impact of Each MRA Dose After TEER Cumulative incidence of the primary outcome was stratified into 4 groups according to the dose of MRA after TEER (A); HR of each MRA dose for the primary outcome were calculated by defining no MRA administration group as a reference (B). ∗*P <* 0.05 by log-rank test; †*P <* 0.05 by Cox proportional HR regression analysis. Abbreviations as in Figure 1.

To further evaluate the prognostic impact of MRA dose following TEER, the patient cohort was stratified into 4 groups based on their MRA dose: 0 mg/d (n = 1,073 [53%]), 12.5 mg/d (n = 139 [7%]), 25 mg/d (n = 581 [29%]), and 37.5 mg/d or greater (n = 233 [12%]). The cumulative incidence of the primary outcome, stratified by these 4 groups, is shown in Figure 4A. Patients who did not receive MRA had a significantly higher incidence of the primary outcome compared with the other 3 groups who received any doses of MRA (*P <* 0.001 for all comparisons). In contrast, the cumulative incidence of the primary outcome did not differ significantly among the groups receiving MRA, regardless of the dose administered *(P =* 0.97).

The HRs for each group are presented in Figure 4B, with the group receiving no MRA serving as the reference. The HRs were below 1.0 for all groups that received any doses of MRA, indicating a lower risk of the primary outcome compared to those who did not receive MRA.

### Subanalysis according to the COPAT profile

In total, 421 patients had severe left ventricular impairment, 18 patients had severe tricuspid regurgitation, 61 patients had severe pulmonary hypertension, and 356 had hemodynamic instability. As a result, 714 (35%) had COPAT-unlike profiles and the other 1,252 (62%) patients had COPAT-like profiles.

In the COPAT-unlike cohort, 372 of 714 (52%) patients received any doses of MRA. The use of any doses of MRA was associated with a significantly lower cumulative incidence of the primary outcome (40% vs 48%; *P =* 0.007). Although it did not reach statistical significance, a higher dose of MRA tended to be associated with a greater cumulative incidence of the primary outcome (≥37.5 mg/d [n = 116] 46%, 25 mg/d [n = 206] 38%, and 12.5 mg/d [n = 51] 31%; *P =* 0.17). The presence of moderate or greater residual MR (n = 175) was not significantly associated with the primary outcome (HR: 0.86; 95% CI: 0.64-1.15; *P =* 0.30).

In the COAPT-like cohort, 546 of 1,252 (44%) patients received any doses of MRA. The use of any doses of MRA was significantly associated with a lower cumulative incidence of the primary outcome (34% vs 42%; *P =* 0.002). The dose of MRA was not significantly associated with the cumulative incidence of the primary outcome (≥37.5 mg/d [n = 111] 30%, 25 mg/d [n = 353] 34%, and 12.5 mg/d [n = 82] 40%; *P =* 0.62). The presence of moderate or greater residual MR (n = 230) was significantly associated with the primary outcome (HR: 1.32; 95% CI: 1.04-1.67; *P =* 0.021).

### Prognostic impact of the change of MRA dose

**Figure 5:**
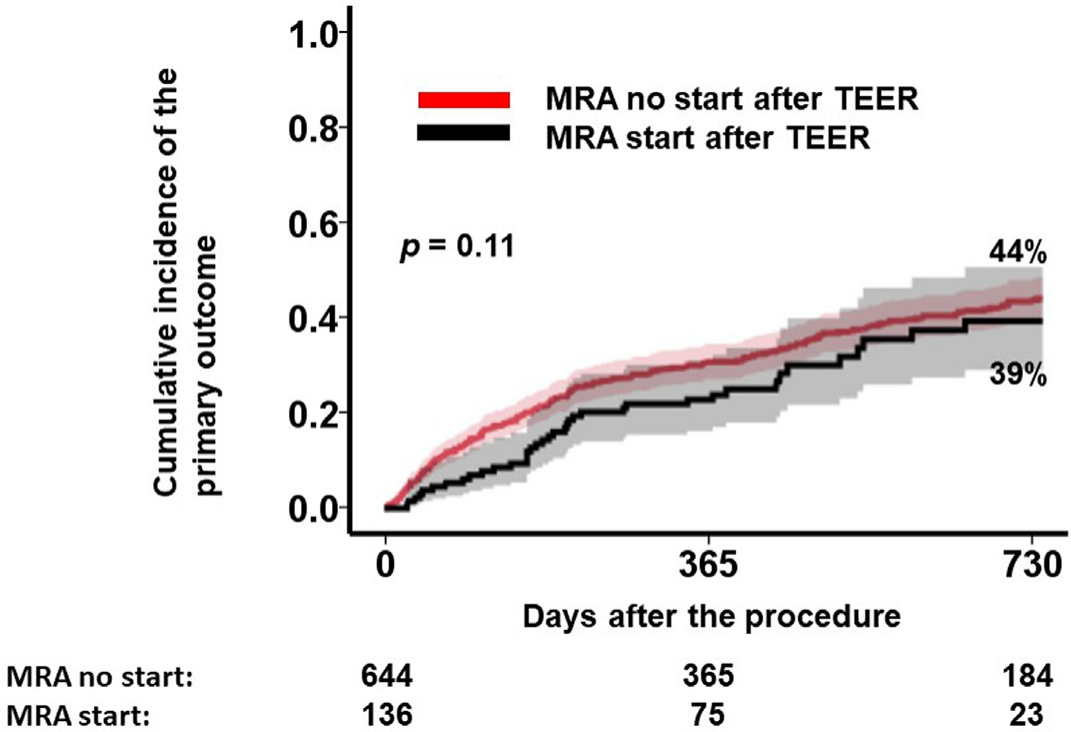
Prognostic Impact of MRA Initiation Following TEER Impact of MRA initiation (vs no MRA initiation) after TEER on the primary outcome was evaluated among the individuals who did not receive MRA before TEER (n = 780). Kaplan-Meier curves were compared by log-rank test. Abbreviations as in Figure 1.

Among the 780 patients who were not receiving MRA therapy before TEER, 136 (17%) patients initiated MRA therapy after TEER for the first time. These patients who started MRA treatment post-TEER tended to have a lower cumulative incidence of the primary outcome compared with those who did not initiate MRA (39% vs 44%; *P =* 0.11) (Figure 5).

A total of 2,011 patients were receiving an MRA dose of <50 mg/d (ie, a submaximal dose) before TEER. Of these, 225 (12%) patients had an increase in their MRA dose after TEER. However, patients who experienced an increase in their MRA dose did not show a significantly lower cumulative incidence of the primary outcome compared with those who did not have an increase in MRA dose (38% vs 41%; *P =* 0.43).

## Discussion

In this retrospective analysis of the OCEAN-Mitral registry, we examined the prognostic impact of the dose of MRA administered after TEER on the 2-year composite outcome of all-cause mortality and heart failure admissions. A total of 2,026 patients with an LVEF of <50% and secondary MR who underwent TEER were included in this registry. Median age of the whole cohort was 77 years, representing the contemporary candidates of TEER, in whom surgical repair was contraindicated. Among them, 953 patients received MRA therapy following TEER. Patients administered an MRA dose of ≥12.5 mg/d post-TEER (ie, any doses of MRA) had an observed 17% reduction in cumulative incidence of death or heart failure readmissions compared with those who received <12.5 mg/d (ie, no MRA). Although the administration of any doses of MRA (even a low-dose MRA) was associated with a reduced risk of the primary outcome compared with no MRA use, further uptitration of the MRA dose did not confer additional benefits in risk reduction (Central Illustration). Furthermore, de novo initiation of MRA or aggressive uptitration of MRA following TEER was not associated with further risk reduction.

**Central Illustration:**
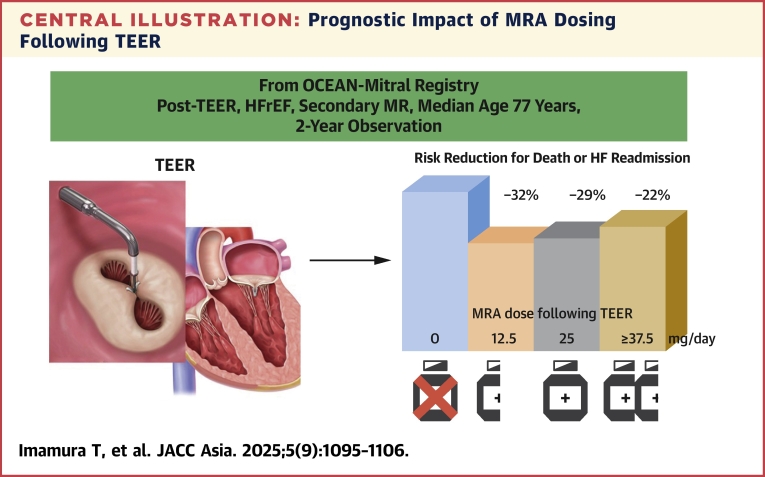
Prognostic Impact of MRA Dosing Following TEER Data on patients with heart failure with reduced ejection fraction (HFrEF) who underwent transcatheter edge-to-edge repair (TEER) for secondary mitral regurgitation (MR) were retrieved from OCEAN-Mitral registry. Mineralocorticoid receptor antagonist (MRA) use was associated with a lower 2-year composite endpoint, whereas dose uptitration was not associated with additional risk reduction. Permission for reuse was obtained from Abbott. HF = heart failure.

### Efficacy and feasibility of MRA administration in older patients receiving TEER

Based on the totality of evidence from major trials such as RALES (Randomized Aldactone Evaluation Study), EPHESUS (Eplerenone Post-Acute Myocardial Infarction Heart Failure Efficacy and Survival Study), and EMPHASIS-HF (Eplerenone in Mild Patients Hospitalization and Survival Study in Heart Failure),^6^, ^7^, ^8^ MRA therapy is well-established as a key component of guideline-directed medical therapy for patients with HFrEF.^1^, ^2^, ^3^ However, these large-scale trials did not include a significant number of older patients. MRA therapy carries the risk of several potential adverse side effects, including hyperkalemia and renal impairment, which may occur at higher rates in older patients with multiple comorbidities. In real-world clinical practice, the prescription rate of MRA, a proven and widely available disease-modifying therapy, remains low with roughly 30% to 40% implementation among heart failure cohorts.^14^

A recent meta-analysis demonstrated that MRAs effectively reduce mortality and morbidity in older patients with heart failure, irrespective of age.^9^ Conversely, worsening renal function was more frequently observed in older patients. Another cohort study, which included 325,263 patients hospitalized for heart failure, found that the prognostic benefit of MRA therapy at discharge was consistent across a wide age range.^15^

Following the encouraging results of the COAPT trial,^12^ a growing number of older patients who are not suitable candidates for surgical intervention are undergoing TEER for secondary MR. Although guideline-directed medical therapy is strongly recommended before TEER, the clinical impact of these medications following TEER remains uncertain. Across various studies, the prescription rate of MRA post-TEER is reported to be around 40% to 50%.^16^, ^17^, ^18^, ^19^

Several studies have investigated the prognostic impact of post-TEER guideline-directed triple therapy, comprising a beta-blocker, renin-angiotensin system inhibitors, and MRAs. A study involving the EuroSMR registry showed that the prescription of this triple therapy was associated with reduced 2-year mortality, irrespective of various comorbidities.^18^ Another study indicated that triple therapy post-TEER led to more substantial reverse remodeling and reduced 2-year mortality.^20^ Additionally, a study using the Japanese nationwide J-MITR registry confirmed that an increasing number of anti-heart failure medications prescribed post-TEER was linked to a lower rate of cardiovascular death and heart failure readmissions.^19^

Few studies have specifically focused on the prognostic impact of MRA therapy following TEER. Our analysis demonstrated that the prescription of MRAs post-TEER was associated with a lower incidence of death and heart failure readmissions, utilizing large-scale, nationwide registry data involving patients with a median age of 77 years—higher than reported in previous studies. Notably, a cutoff of MRA dose to achieve the primary outcome was calculated as 12.5 mg/d, indicating that even a low-dose MRA had a significant prognostic impact compared with no MRA use. The success of MRA prescription is largely influenced by comorbidities, such as renal function. Notably, the prognostic benefit of MRA use remained significant even after accounting for other factors that may alter the primary outcome of interest.

### Impact of high-dose MRA following TEER

Current guidelines recommend uptitrating the dose of anti–heart failure medications, including MRAs, to the maximum tolerated dose in patients with HFrEF.^1^, ^2^, ^3^ Specifically, in the RALES trial, uptitration to 50 mg of spironolactone was recommended after 8 weeks on the 25 mg dose if patients had ongoing symptoms of heart failure notwithstanding clinically significant hyperkalemia. In a phase II trial involving heart failure patients, urinary aldosterone concentrations were found to increase following the administration of eplerenone in a dose-dependent manner; however, further increases in MRA doses were not associated with additional reductions in plasma B-type natriuretic peptide levels. In another epidemiological study involving 3,195 patients with chronic kidney disease, whose mean age was 66 years, the association between MRA use and renal replacement therapy inhibition was dose-dependent.^21^ Although the clinical benefits of beta-blocker dose uptitration are well established, the implications of MRA uptitration remain controversial.

In this study, we found that increasing the dose of MRA beyond 12.5 mg/d did not lead to further improvements in mortality or morbidity in patients undergoing TEER. In the prior study utilizing the EuroSMR registry, titration of heart failure triple therapy, including MRA, to at least one-half of the target dose after TEER was associated with improved clinical outcomes, particularly among patients with severe right ventricular dysfunction.^18^ However, the analysis did not evaluate the prognostic impact of dose escalation beyond one-half of the target dose. Additionally, the study did not specifically focus on MRA, which is often the most challenging component of guideline-directed medical therapy to uptitrate because of its associated adverse effects.

In the subanalysis of our present study, higher doses of MRA trended to correlate with worse clinical outcomes in the COAPT-unlike cohort, in whom the benefit of successful TEER might be limited. This finding suggests that while MRA uptitration may offer benefits in specific subgroups, such as patients with right ventricular failure, it could potentially be harmful in other cohorts, likely caused by a worse baseline clinical profile or increased vulnerability to adverse effects. These observations highlight the necessity of tailoring therapeutic strategies to individual patient characteristics to optimize post-TEER outcomes in the future.

### Consideration for MRA dosing

In the present study, the administration of a low-dose MRA after TEER was associated with improved clinical outcomes compared with no MRA use. However, increasing the MRA dose beyond a low dose did not confer clinically significant additional benefits. Initiating MRA therapy following TEER was linked to improved clinical outcomes, while further dose uptitration failed to provide extra clinical advantages.

Optimization of medical therapy with maximally tolerated, evidenced-based, guideline-directed medical therapy in patients with HFrEF must be undertaken before consideration of TEER. Nonetheless, the improvement in MR achieved through TEER might offer an additional opportunity to initiate and maximize MRA treatment along with other heart failure–specific therapies, because of hemodynamic stabilization and the subsequent improvement in end-organ dysfunction.^22^ Based on our findings, in those who were not receiving MRA before TEER, implementation postintervention should strongly be considered. Notably, even a low-dose MRA is welcome rather than no MRA use. We should be careful of further dose uptitration of MRA given its dose-dependent adverse effects and few additional clinical benefits.

### Study limitations

Although we investigated the prognostic impact of MRA dosing, the retrospective nature of the study precludes definitive conclusions regarding causality. Importantly, we did not collect comprehensive echocardiographic and laboratory data following TEER, which would be necessary to further elucidate the detailed long-term clinical effects of different MRA doses. Although we made extensive efforts to adjust for potential confounders, the influence of these factors on our primary findings cannot be entirely ruled out. Additionally, while the dosing of MRA in Japanese patients is considered comparable to that in European and North American populations,^1^, ^2^, ^3^ the generalizability of our findings requires validation through further international studies. We are not against the guideline-recommended strategy to uptitrate the dose of MRA. Our finding is restricted to the older patients receiving TEER. The minimum dose of MRA in the present study was 12.5 mg/d. The prognostic impact of extremely low dose of MRA (ie, below 12.5 mg/d) vs no MRA use remains uncertain.

## Conclusions

In older patients undergoing TEER for secondary MR associated with systolic heart failure, the administration of any doses of MRA was associated with improved clinical outcomes compared to no MRA therapy. The study also revealed that increasing the MRA dose beyond the low dose did not result in further significant improvements in clinical outcomes. This suggests that although MRA therapy is beneficial, its effects may reach a plateau at lower doses, and further dose escalation may not provide additional clinical advantages. Future research is needed to understand the mechanisms underlying the observed dose-response relationship and validate these findings in diverse populations to ensure their generalizability.

## Funding Support and Author Disclosures

The OCEAN-Mitral registry, which is part of the OCEAN-SHD registry, is supported by Edwards Lifesciences, Medtronic Japan, Boston Scientific, Abbott Medical Japan, and Daiichi-Sankyo Company. Drs. Kubo, Saji, Izumo, Watanabe, and Amaki are clinical proctors of transcatheter edge-to-edge repair for Abbott Medical; and have received consultant fees from Abbott Medical. Dr Asami is a clinical proctor of transcatheter edge-to-edge repair for Abbott Medical; and has received speaker fees from Abbott Medical. Dr Kodama has received speaker fees from Abbott Medical. Dr. Yamamoto is clinical proctor of transcatheter edge-to-edge repair for Abbott Medical; and has received lecture fees from Abbott Medical. Dr Yamaguchi is a clinical proctor of transcatheter edge-to-edge repair for Abbott Medical; and has received a lecture fee and scholarship donation from Abbott Medical. Dr Ohno has received consultant, advisor, and speaker fees from Abbott Medical. Drs. Enta, Shirai, Mizuno, Ueno, Mizutani, Bota, and Hayashida are clinical proctors of transcatheter edge-to-edge repair for Abbott Medical. All other authors have reported that they have no relationships relevant to the contents of this paper to disclose.
